# Optimizing prognostic factors of five-year survival in gastric cancer patients using feature selection techniques with machine learning algorithms: a comparative study

**DOI:** 10.1186/s12911-023-02154-y

**Published:** 2023-04-06

**Authors:** Mohammad Reza Afrash, Esmat Mirbagheri, Mehrnaz Mashoufi, Hadi Kazemi-Arpanahi

**Affiliations:** 1 Department of Artificial Intelligence, Smart University of Medical Sciences, Tehran, Iran; 2 Department of Health Information Management, School of Health Management and Information Sciences, Iran University of Medical Sciences, Tehran, Iran; 3 Department of Health Information Management, Ardabil University of Medical Sciences, Ardabil, Iran; 4 Department of Health Information Technology, Abadan University of Medical Sciences, Abadan, Iran

**Keywords:** Feature selection, Gastric cancer survival, Machine learning, Benchmarking

## Abstract

**Background:**

Gastric cancer is the most common malignant tumor worldwide and a leading cause of cancer deaths. This neoplasm has a poor prognosis and heterogeneous outcomes. Survivability prediction may help select the best treatment plan based on an individual’s prognosis. Numerous clinical and pathological features are generally used in predicting gastric cancer survival, and their influence on the survival of this cancer has not been fully elucidated. Moreover, the five-year survivability prognosis performances of feature selection methods with machine learning (ML) classifiers for gastric cancer have not been fully benchmarked. Therefore, we adopted several well-known feature selection methods and ML classifiers together to determine the best-paired feature selection-classifier for this purpose.

**Methods:**

This was a retrospective study on a dataset of 974 patients diagnosed with gastric cancer in the Ayatollah Talleghani Hospital, Abadan, Iran. First, four feature selection algorithms, including Relief, Boruta, least absolute shrinkage and selection operator (LASSO), and minimum redundancy maximum relevance (mRMR) were used to select a set of relevant features that are very informative for five-year survival prediction in gastric cancer patients. Then, each feature set was fed to three classifiers: XG Boost (XGB), hist gradient boosting (HGB), and support vector machine (SVM) to develop predictive models. Finally, paired feature selection-classifier methods were evaluated to select the best-paired method using the area under the curve (AUC), accuracy, sensitivity, specificity, and f1-score metrics.

**Results:**

The LASSO feature selection algorithm combined with the XG Boost classifier achieved an accuracy of 89.10%, a specificity of 87.15%, a sensitivity of 89.42%, an AUC of 89.37%, and an f1-score of 90.8%. Tumor stage, history of other cancers, lymphatic invasion, tumor site, type of treatment, body weight, histological type, and addiction were identified as the most significant factors affecting gastric cancer survival.

**Conclusions:**

This study proved the worth of the paired feature selection-classifier to identify the best path that could augment the five-year survival prediction in gastric cancer patients. Our results were better than those of previous studies, both in terms of the time required to form the models and the performance measurement criteria of the algorithms. These findings may be very promising and can, therefore, inform clinical decision-making and shed light on future studies.

## Introduction

According to Global Cancer Observatory (GLOBOCAN) statistics, gastric cancer accounts for 5.6% of all new cancer cases (1,089,103 cases), ranking fifth in terms of incidence. This cancer accounts for 768,793 deaths worldwide and about 7.7% of all cancer-related deaths, ranking fourth in this regard [1]. Contrary to the globally declining trend of gastric cancer during the last few decades, in many Asian countries including Iran, this cancer is still on the rise in terms of incidence and mortality rates. Based on the GLOBOCAN 2020 report, gastric cancer is the second most common cancer in Iran with 13,191 new cases (11.2%) of all cancers, and ranks first among all cancer-related deaths with 79,136 (16.4%) deaths. This increase in incidence in Iran is probably due to the recent demographic and epidemiological changes in the Iranian population [2, 3].

An important problem faced by patients with gastric cancer, like other clinical fields, is the multidimensional and ambiguous diagnostic and treatment processes [4]. The treatment of gastric cancer depends largely on the judgment of prognosis, which strongly depends on the stage at which it is diagnosed [5, 6]. The five-year relative survival rate for lesions is up to 70% in the early stages and up to 4% in the advanced stages [5, 7, 8]. Survival often refers to a patient’s chance of surviving up to five years after a cancer diagnosis. This index is usually used in medicine to evaluate the effectiveness of surgical and therapeutic plans [9]. Accurately estimating the survival of patients with gastric cancer can help doctors reach better verdicts about the diagnosis and treatment process, including the choice of treatment methods, treatment schedules, and follow-up visits, thereby improving patient outcomes and reducing costs [10, 11].

Accurate prediction of the gastric cancer outcome prognosis is the basis for customizing and personalizing treatment protocols [12]. Prognosis estimation using conventional statistical methods is very difficult because patient characteristics have multidimensional and non-linear relationships. Therefore, to personalize care and treatment programs, computational approaches such as machine learning (ML) models are used as they can analyze these multidimensional and complex features via multiple processing layers, including complex structures or multiple nonlinear transformations [13–15].

Currently, with the advent of advanced technologies, a great amount of high-dimensional data (several features with various types of values) has been produced in medicine, especially in domains related to cancer care and treatment processes [16, 17]. The high dimensions of data and quality-related problems such as irrelevant, missing, duplicate, useless, and misleading features make it more problematic to gain insights from data [18]. In addition, if the sample size is small and there are numerous variables, problems related to overfitting may arise. This problem happens when the number of coefficients exceeds the number of observations [19, 20].

High-dimensional medical data decreases the efficiency of computational models [21]. A simple prediction model with optimized features known as parsimonious achieves good performance compared to a full-featured, highly complex model [22]. Therefore, raw and high-dimensional data should be preprocessed to make the data fitter for further analysis. Due to the vast number of clinicopathologic variables and the small sample size, it is important to implement feature selection methods in the proposed model to overcome some of these problems and avoid overfitting [23].

Feature selection is an important stage of data preprocessing for reducing data dimensionality [24]. Selecting effective features for the best model fitting in ML algorithms is a difficult task. It is a critical step in the analysis of complex and multidimensional data to select the best features before building a predictive model [23]. Identifying relevant features helps reduce unnecessary, redundant, and noisy features which, in turn, provides faster and better computational results [25]. Especially for the analysis of high-dimensional datasets, ignoring irrelevant and redundant features often helps improve predictive performance, computation time, and comprehensibility. This can be achieved by selecting a set of important and influential features on the target variable. Due to the large number of feature selection methods available, benchmarking studies are of great importance to identify the best methods to use in data analysis [20].

Choosing the appropriate feature selection method for a specific scenario is not a trivial task; therefore, several strategies have been investigated to classify unpaired feature selection methods. The most widely used classification strategy classifies methods into filter, wrapper, and embedded based on ensemble classifiers. The filter method splits up feature selection from classifier construction and assesses feature relevance based only on the data’s intrinsic properties [26, 27], often applied to the analysis of high-dimensional data (such as microarray data). The wrapper method assesses the classification performance of selected features and continues to search/optimize until a certain accuracy criterion is met [28, 29]. Besides using each feature selection method individually, ensemble feature selection is created by integrating several methods into one algorithm. It has the most prominent ability to address stability problems that are typically poor by existing feature selection methods, assuming that the output of multiple models is better than that of any individual model [30]. However, conventional feature selection techniques have some limitations. These methods depend only on accuracy as a metric for evaluation. Moreover, due to the inherent characteristics of medical data, such as vague, imbalanced, and inaccurate data, a highly misleading accuracy is attained, eventually providing false risk prediction. Thus, accuracy alone is not a sufficient criterion for evaluation. Therefore, while emphasizing the use of the area under the curve (AUC) along with accuracy to achieve a robust prediction model, recent studies have proposed novel feature selection models such as novel feature reduction (NFR) and advanced hybrid ensemble gain ratio feature selection (AHEG-FS) to overcome the abovementioned limitations [31, 32].

Despite the high incidence of gastric cancer in Iran, there was no reliable study to determine the survival risk factors of the disease using feature selection methods. Thus, the current study was conducted to contribute to the prediction of five-year survival by identifying important features and their complex effects on gastric cancer patients using four feature selection methods with three classification algorithms.

## Methods

### Study design and settings

**Fig. 1:**
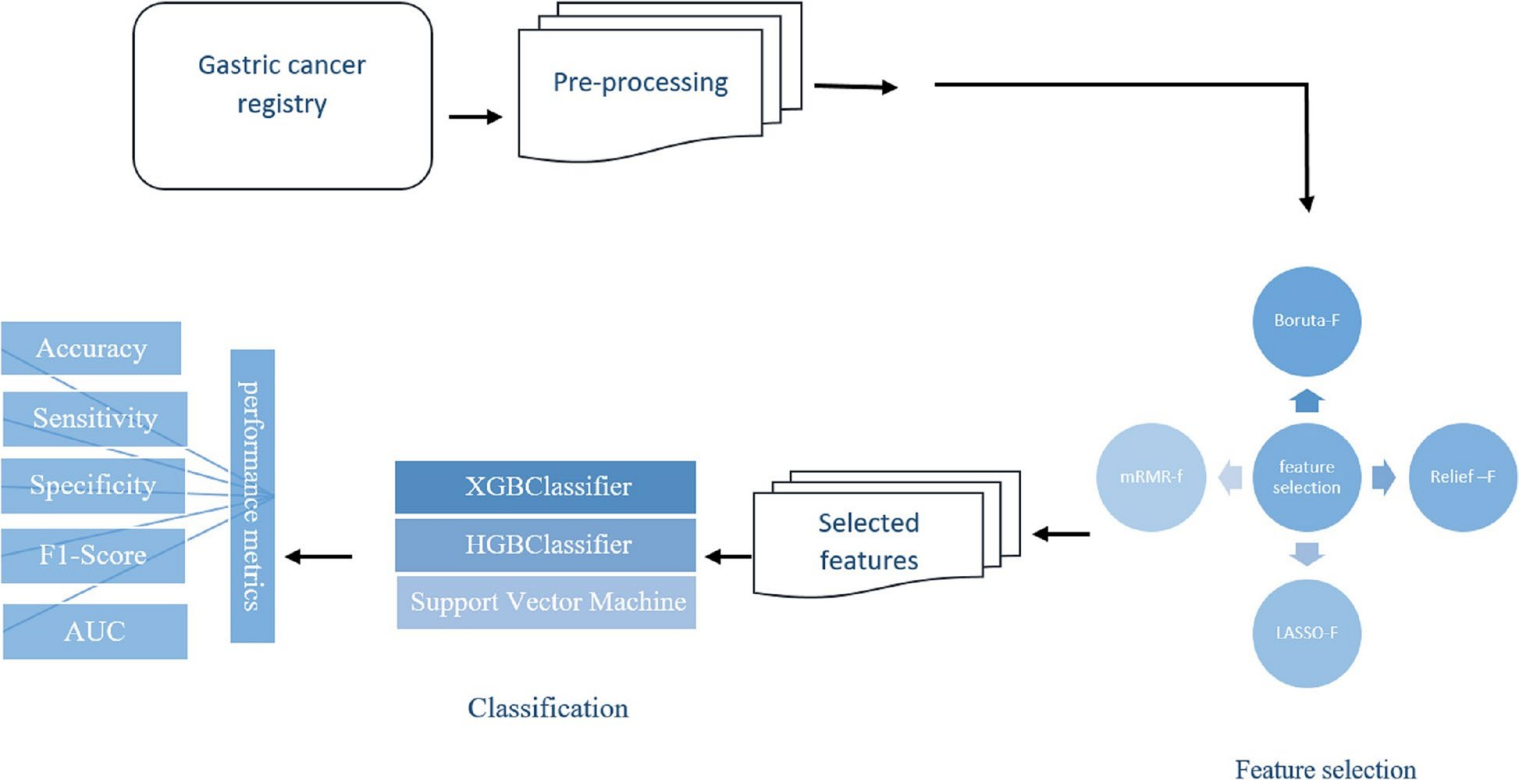
The framework of gastric cancer five-year survival prediction

This was a retrospective study using a single-center registry database conducted in 2022 to predict the most important features of gastric cancer survival. In this section, an overall explanation is presented to develop an intelligent ML-based system over the dataset of patients with gastric cancer. We first describe the dataset used in this research. Then, the feature extraction and the feature selection procedures are introduced. Next, we describe the applied ML algorithms. Finally, we provide the implementation details of our proposed models. The structure of the proposed method is depicted in Fig. 1.

### Data collection

The data of 1220 patients diagnosed with gastric cancer in the Cancer Research Center of Ayatollah Talleghani Hospital, Abadan, Iran (2010–2017) were used. We retrospectively reviewed the demographics, clinical information history, and treatment data of the patients until death or until the data registered on the last follow-up. Adult patients (> 18 years) who had received a histopathological diagnosis of gastric adenocarcinoma, had a regular follow-up, and had a Karnofsky Performance Scale (KPS) score of 70 were included. From 1220 patient records, 59 records of patients who were aged < 18 years old were excluded. In the preprocessing phase, 187 incomplete rows of data (with missing data of greater than 70%) were removed. After these criteria were applied, a total of 974 patients (399 survived and 575 passed away within 5 years) remained for additional analyses.

Several meetings were held with several oncologists to remove unnecessary variables that were considered less significant prognostic variables for gastric cancer survival. The Health Research and Ethics Committee of the Abadan University of Medical Sciences approved this study, and all the participants provided written informed consent (IR.ABADANUMS.REC.1401.065).

### Feature extraction

Feature extraction is a crucial step in the data mining workflow. It is an approach to extracting a set of variables from the original dataset and usually reduces the feature space. The main aim of feature extraction is to capture the most important features from the original dataset and represent the information of these newly extracted features in a lower-dimensionality dataset. Herein, 28 variables were extracted from the original dataset (Table 1).

**Table 1:** Characteristics of patients with gastric cancer

Code	Features Name	Scale	Value
1	Sex	Nominal	Male – Female
2	Age at diagnosis	Interval	Ranged between 23 to 79
3	Body weight	Interval	> > 60, < 60
4	Weight loss	Nominal	Yes – No
5	Addiction	Nominal	Yes – No
6	History of another cancer	Nominal	Yes – No
7	Family history of gastric cancer	Nominal	Yes – No
8	Family history of other cancer	Nominal	Yes – No
9	Tumor size	Ordinal	< < 3 CM, 3–6 CM, > 6
10	Tumor stage	Ordinal	IA, IB, IIA, IIB, IIIA, IIIB, IIIC
11	Tumor site	Ordinal	Lower third, Middle third, Upper third, Whole stomach
12	Metastatic status	Nominal	Yes – No
13	Histological type/ Histology	Ordinal	Rivers, Diffuse, Complex
14	Lymphatic invasion	Nominal	Positive, Negative
15	Vascular invasion	Nominal	Positive, Negative
16	Histopathology type	Ordinal	Adenocarcinoma, Lymphoma, Sarcoma
17	Treatment	Ordinal	Surgery, Chemotherapy, Surgery + Chemotherapy + Radiotherapy
Outcome variable		Nominal	Death, Alive

### Feature selection

In data science and learning, the individual operator usually chooses potentially important variables. However, not all these variables may be related to the goals of learning, and some of them may be unimportant, redundant, and noisy. Such variables can be chosen by automated ML feature selection approaches. Feature selection methods evaluate the relevance of a variable or a set of variables based on given goals.

The advantages of feature selection are:

- Fewer computational requirements
- Improved understanding of the problem
- Building better generalizable models
- Avoiding the long running time of ML models
- Providing faster and more cost-effective ML models

There are three categories of feature selection algorithms applied in the literature, namely, filter, wrapper, and embedded techniques [25, 33].

#### The filter approach

**Fig. 2:**
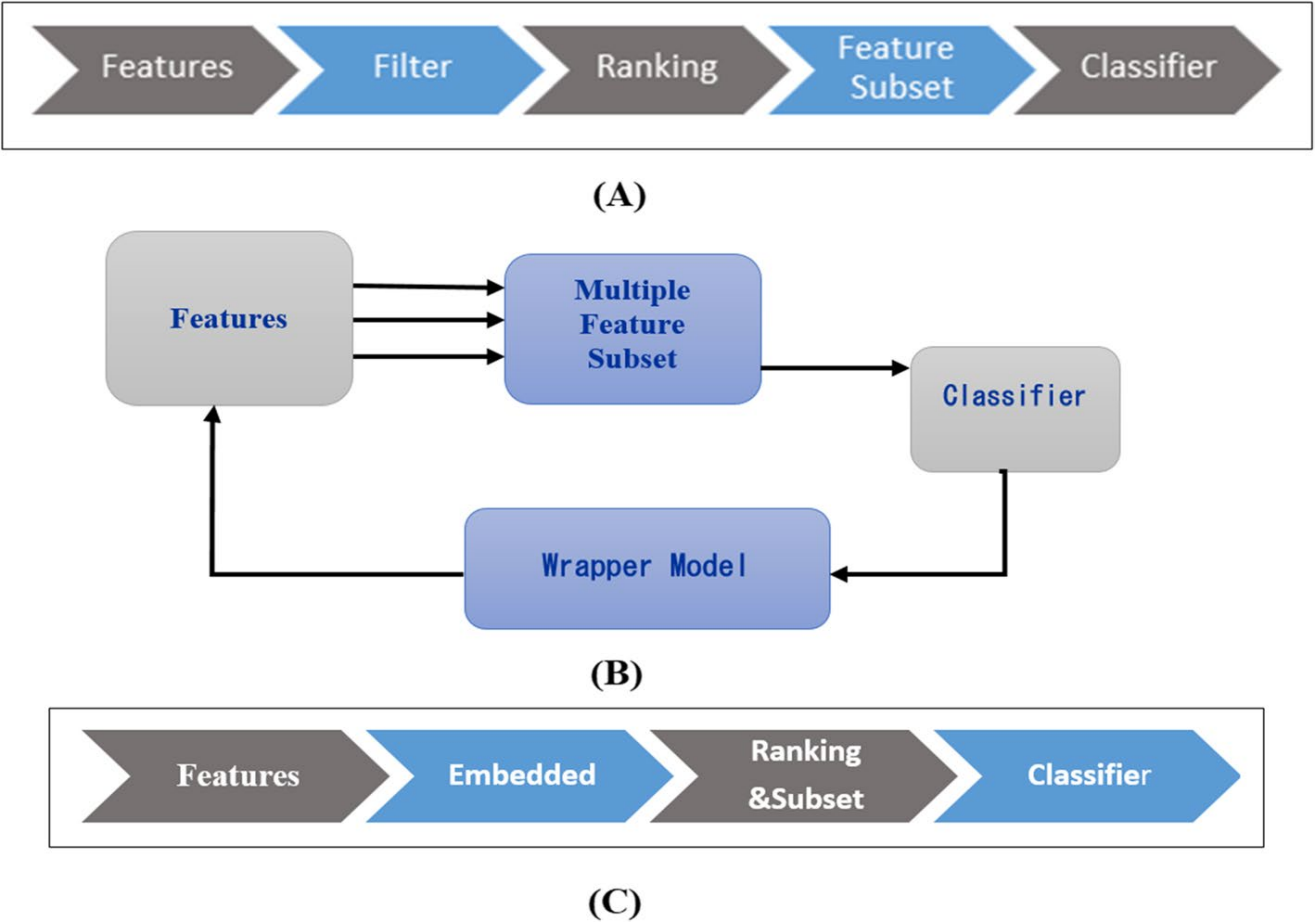
The process of embedded feature selection types

The filter approach selects variables based on four evaluation metrics of distance, information, dependency, and consistency (statistical assumptions). Figure 2 depicts the process of the filter feature selection models (Part A).

#### The wrapper models

The wrapper models select the most ideal variables based on the performance of an ML classification algorithm in a given subset of variables distinguished by a search technique. The wrapper calculates the accuracy of the classifier for each variable that can be added to or deleted from the variable’s subset. Figure 2 displays the process of wrapper feature selection models (Part B).

#### The embedded techniques

The embedded techniques bridge the gap between filter and wrapper methods. First, they use measurable and statistical metrics such as a filter to choose some variables; then, using a classifier, they chose the subset with the highest classification accuracy. Figure 2 illustrates the process of embedded feature selection models (Part C).

We used four feature selection methods, namely, (a) Relief, (b) minimal-redundancy-maximal-relevance (mRMR), (c) least absolute shrinkage and selection operator (LASSO), and (d) Boruta. The types of feature selection approaches used in this study are shown in Table 2.

**Table 2:** Types of feature selection methods used in the present study

N	Feature selection	Type
1	Relief	Filter
2	mRMR	Filter
3	LASSO	Embedded
4	Boruta	Wrapper

##### Relief

Relief is a well-known technique in the categories of filter feature selection. The main idea is to assign a weight scale to all features that can then be used to rank and choose the highest-scoring variables for feature selection. Highly important features to the goal have large weights, while the remaining features have low weights. Relief uses the same approaches as those in the K-Nearest neighbor algorithm that calculates the weight of each feature. That is beneficial when assessing parameters with interdependencies and noisy datasets.

##### mRMR

mRMR is a filter feature selection method that uses a heuristic search method to select optimum variables with maximum relevance and minimum redundancy. This approach can successfully decrease redundant variables while retaining the features that are important and relevant for the classifier. This method uses mutual information to calculate the relevant or redundant feature.

##### LASSO

LASSO is an embedded-based technique that selects the most relevant features based on updating the absolute value of the feature coefficient. Some feature coefficients of variables become zero, and these features with zero coefficients are deleted from the variable subset. LASSO shows a good performance in cases with low feature coefficients. The variables with high coefficients will be included in the select variable subsets. In a case with a high correlation value, some irrelevant features may be included in the feature subset.

##### Boruta

Boruta is a wrapper-based technique for feature selection that is based on a non-parametric algorithm (RF algorithm). This approach finds relevant features by comparing the significance of original variables with the importance of their randomly shuffled copies and selects the features with greater importance than their shuffled copies. These copies of the variables are called shadow variables which are added to the original data set, but they miss the connection with the dependent features. Then, the importance of the original features in the created random forests algorithm is measured to that of the shadow features to determine the importance of the original features. The Z score is used to measure the importance of features. Features that have scored higher than the uppermost Z score between shadow features are tagged as important features.

### Model development and performance evaluation

Three ML models were developed to select the most important feature set gastric cancer survival among the patients. Prediction models, including eXtreme Gradient Boosting (XGBoost), Hist Gradient Boosting classifier (HGB), and Support Vector Machine (SVM) were established by ML to assess each feature set. Initially, 90 of the datasets were randomly selected to train the classifiers, and the remaining Sect. (10%) was used for testing the models. The K-fold cross-validation method and hyperparameter tuning were used to reduce overfitting and enhance the performance of the models. Finally, after classifier training, the performance of the trained classifier was calculated in terms of the average metrics, including accuracy, sensitivity, specificity, f1-score, and the average AUC on the test set (Eqs. 1 to 6). Confidence intervals (95 CI) and classifier performance metrics were computed. To develop the prediction models, modeling was performed on an HP laptop using Microsoft Windows 10 with an Intel(R) CPU core i7, 2.40 GHz, and 8-GB RAM. Python 3.8.1 was used to develop the machine learning models. Scikit-Learn was utilized to develop the ML models, and Pandas Libraries were utilized to analyze the data correlations. All libraries were open-source.

1. $$\text{classification satisfactory}=\frac{\mathrm{TP}+\mathrm{TN}}{\mathrm{TP}+\mathrm{TN}+\mathrm{FP}+\mathrm{FN}}\ast100$$ 
2. $$\text{classification sensitivity}=\frac{\mathrm{Tp}}{\mathrm{TP}+\mathrm{FN}}\ast100$$ 
3. $$\text{classification specificity}=\frac{\mathrm{TN}}{\mathrm{TN}+\mathrm{FP}}\ast100$$ 
4. $$\text{classification error}=\frac{\mathrm{FP}+\mathrm{FN}}{\mathrm{TP}+\mathrm{TN}+\mathrm{FP}+\mathrm{FN}}\ast100$$ 
5. $$\mathrm{f}-\mathrm{measure }=2 \frac{\mathrm{precision}*\mathrm{sensitivity }}{\mathrm{precision}+\mathrm{ sensitivity}}$$

## Results

### Characteristics of the participants

A total of 974 patients with gastric cancer met the predetermined inclusion criteria. Their age ranged from 23 to 79, with an average age of 57.25 years. Moreover, 648 (66.53%) patients were male and 326 (33.47%) were women. Of these, 399 (40.96%) patients survived and 575 (59.04%) passed away. The distribution of demographic, epidemiological, and clinical variables of gastric cancer patients is shown in Table 3.

**Table 3:** Characteristics of patients with gastric cancer

N	Features Name	Classifications	Total	Survived	Did not survived
				**N**	**N**
1	Age at diagnosis	< 45	233	197	36
		> > 45	741	483	258
2	Sex	Female	326	218	108
		Male	648	462	186
3	Body weight	< 60	263	174	89
		> > 60	711	506	205
4	Weight loss	Yes	369	231	138
		No	605	449	156
5	Addiction	Yes	117	47	70
		No	857	506	351
6	History of other cancers	Yes	155	74	81
		No	819	606	213
7	Family history of gastric cancer	Yes	23	7	16
		No	951	673	278
8	Family history of other cancers	Yes	62	27	35
		No	912	653	259
9	Tumor size	<< 3 CM	326	269	57
		3–6 CM	459	324	135
		> 6	189	87	102
10	Tumor stage	IA	43	31	12
		IB	134	107	27
		IIA	149	117	32
		IIB	190	149	41
		IIIA	177	126	51
		IIIB	129	47	82
		IIIC	152	83	69
11	Tumor site	Lower third	315	288	27
		Middle third	340	256	84
		Upper third	284	132	152
		Whole stomach	35	4	31
12	Metastatic status	Yes	227	93	134
		No	549	437	112
		Unknown	198	150	48
14	Lymphatic invasion	Positive	642	433	209
		Negative	332	247	85
15	Vascular invasion	Positive	583	364	219
		Negative	391	316	75
16	Histopathology type	Adenocarcinoma	670	507	163
		Lymphoma	146	98	48
		Sarcoma	158	75	83
17	Type of treatment	Surgery	192	75	117
		Chemotherapy	366	292	74
		Surgery + Chemotherapy + Radiotherapy	416	313	103
Outcome		Alive	974	399	575
		Death			

### Result of feature selection methods

Before feeding the dataset into the classifiers, we used four well-known feature selection methods to select the most important features for gastric cancer survival prediction. The weight or importance score of each variable selected from the full-featured dataset was identified using four feature selection methods. In the following, the results of the performed feature selection algorithms in terms of the selected variables and their ranks are described.

#### Result of relief feature selection algorithm

**Fig. 3:**
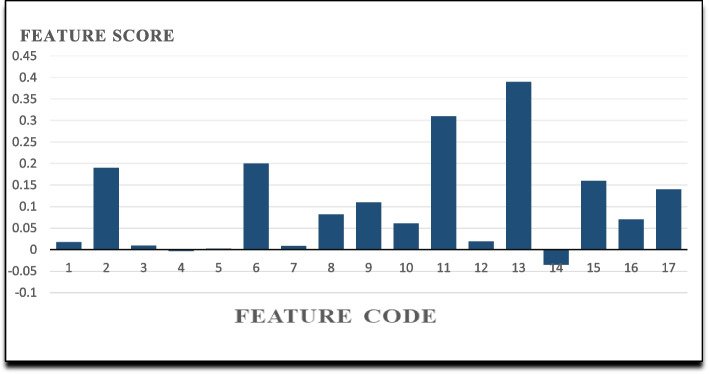
Important features’ scores selected by the Relief feature selection algorithm

The Relief algorithm selects features based on their weights. Eight important features were ranked by the Relief algorithm. According to this rank, the most important features for predicting the survival of gastric cancer chosen by Relief are given in Fig. 3.

#### Results of mRMR feature selection algorithm

**Fig. 4:**
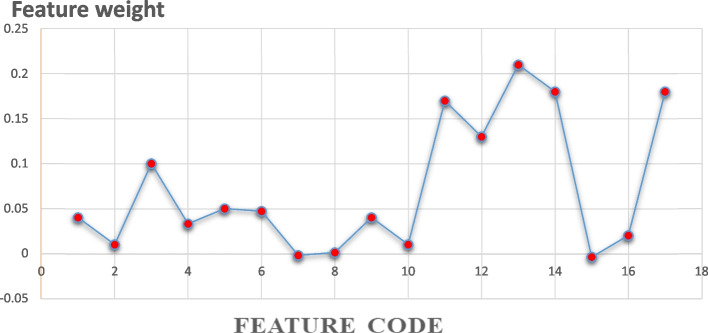
Feature weights based on the mRMR feature selection algorithm

mRMR selects highly related features based on mutual information. The eight highly related features based on mRMR are ranked in Fig. 4.

The eight most important input variables for predicting gastric cancer survival selected by mRMR were tumor stage, history of other cancers, lymphatic invasion, tumor site, type of treatment, body weight, histological type, and addiction.

#### Results of the LASSO feature selection algorithm

**Fig. 5:**
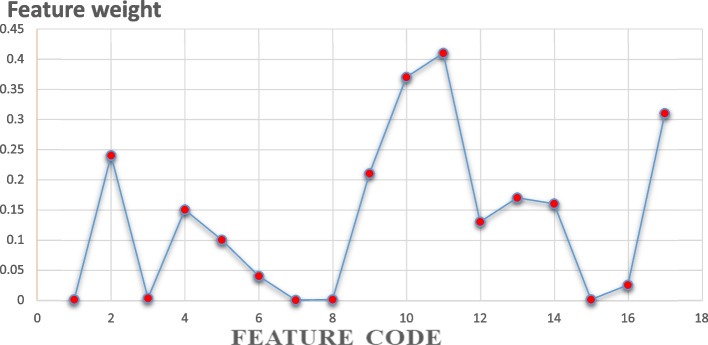
Feature weights based on the LASSO feature selection algorithm

LASSO ranks features based on updating the absolute value of the features’ coefficients. The eight most important variables chosen by LASSO are represented in Fig. 5.

#### Results of the Boruta feature selection algorithm

**Fig. 6:**
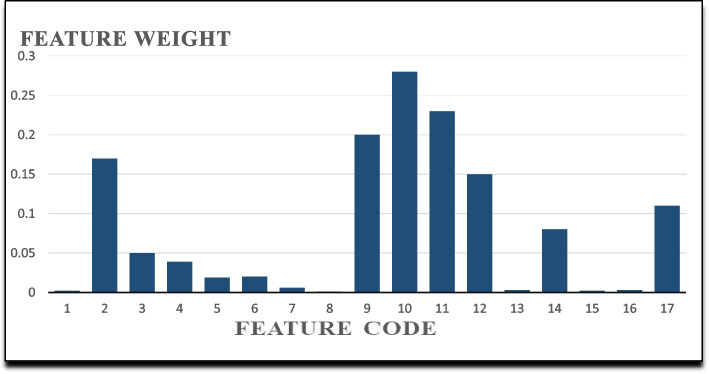
Feature weights based on the Boruta feature selection algorithm

Boruta works based on a random forest classifier. Figure 6 depicts the importance of features selected by the Boruta algorithm. Based on these importance scores, the most important features in gastric cancer survival prediction are ranked in Table 4.

**Table 4:** Features selected by feature selection algorithms and their ranks

Rank	Boruta	mRMR	Relief	LASSO
1	Tumor stage	Tumor stage	Tumor site	Histological type
2	Tumor site	History of other cancers	History of other cancers	Tumor site
3	Tumor size	Lymphatic invasion	Tumor stage	History of other cancer
4	Age	Tumor site	Type of Treatment	Age
5	Metastatic status	Type of Treatment	Tumor size	Vascular invasion
6	Type of treatment	Body weight	Lymphatic invasion	Tumor size
7	Lymphatic invasion	Histological type	Weight loss	Type of Treatment
8	Body weight	Addiction	Metastatic status	Tumor stage

Based on the results (Table 4), the most significant variables for predicting survival among patients with gastric cancer were tumor stage, tumor site, tumor size, and history of other cancers, which were ranked from 1 to 3 by all the feature selection algorithms. Age, vascular invasion, type of treatment, weight loss, metastatic status, addiction, and lymphatic invasion were selected by one or more feature selection algorithms. More details about these variables can be found in Table 4.

### Results of hyperparameter tuning

The hyperparameter tunings of the prediction models were optimized for better prediction during model testing. Table 5 represents the optimized hyperparameters of the classifiers for gastric cancer survival prediction based on the selected feature subset.

**Table 5:** Best hyperparameters selected to be fed into the classifiers

Num	ML Models	Hyper-parameters	F1-score
1	HGB classifier	(‘verbose’:2,’random_state’:999,’n_estimators’:14,’max_deph’:7’criterion’: gini’)	81.32
4	SVM (kernel = RBF)	C = 15, G = 0.004	76.14
5	XG Boost Classifier	‘min_chid_weigh’ = 1’max_depht’ = 16,’learning_rate’ = 0.4, ‘gamma’ = 0.1, ‘colsample_bytree’ = 0.4	83.7

### Performance of classifiers

This section represents the results of the three ML classifiers applied to the full-features dataset and selected-features subset by four feature selection algorithms, namely, Boruta, mRMR, Relief, and LASSO (Table 6).

**Table 6:** Performance evaluation of the selected ML algorithm

N	FS algorithm	FS Type	Feature set	Classifier	Performance metrics						
						**Accuracy**	**Sensitivity**	**Specificity**	**F1-score**	**AUC**	**Time to build a model (s)**
**1**	Without performing feature selection	NONE	Full-featured dataset	SVM		69.47	70.31	69.13	70.23	70.37	1635
					95% CI	(0.71, 0.69)	(0.73, 0.68)	(0.71, 0.68)	(0.71, 0.68)	(0.71, 0.68)	
				HGB		62.58	62.72	61.63	62.18	62.06	1241
					95% CI	(0.64, 0.61)	(0.64, 0.61)	(0.61, 0.60)	(0.63, 0.61)	(0.63, 0.61)	
				XGB		68.25	66.82	71.63	69.23	69.14	690
					95% CI	(0.69, 0.67)	(0.69, 0.67)	(0.73, 0.70)	(0.72, 0.69)	(0.71, 0.68)	
2	Boruta-F	Wrapper-based technique	Tumor stage, tumor site, tumor size, age, metastatic status, type of treatment, lymphatic invasion, body weight	SVM		85.68	86.54	86.39	85.64	83.77	1419
					95% CI	(8.401, 8.715)	(8.520, 8.795)	(8.571, 8.743)	(8.421, 8.815)	(8.274, 8.435)	
				HGB		88.25	89.71	86.13	89.31	88.63	1360
					95% CI	(8.72, 8.947)	(8.811, 9.145)	(8.531, 8.729)	(8.80, 9.024)	(8.631, 8.985)	
				XGB		82.54	86.43	87.02	85.97	86.10	730
					95% CI	(8.167, 8.346)	(8.517, 8.812)	(8.60, 8.827)	(8.42, 8.62)	(8.537, 8.750)	
3	mRMR-F	Filter feature selection method	Tumor stage, history of other cancers, lymphatic invasion, tumor site, type of treatment, body weight, histological type, addiction	SVM		82.12	83.42	81.24	82.98	83.15	1752
					95% CI	(8.094, 8.327)	(8.251, 8.491)	(8.02, 8.8217)	(8.147, 8.410)	(8.192, 8.551)	
				HGB		81.46	81.42	81.62	80.52	80.14	1502
					95% CI	(8.094, 8.327)	(8.251, 8.491)	(8.02, 8.8217)	(8.147, 8.410)	(8.192, 8.551)	
				XGB		80.24	80.52	80.35	80.26	81.24	1489
					95% CI	(7.927, 8.192)	(7.974, 8.251)	(7.914, 8.241)	(7.915, 8.15)	(8.037, 8.301)	
4	LASSO-F	Embedded-based technique	Tumor site, tumor stage, age, type of treatment, tumor size, lymphatic invasion, weight loss, metastatic status	SVM		83.07	85.21	82.49	83.75	81.59	950
					95% CI	(8.19, 8.51)	(8.420, 8.725)	(8.14, 8.397)	(8.17, 8.496)	(8.052, 8.30)	
				HGB		84.12	84.62	83.19	82.45	83.09	1037
					95% CI	(8.274, 8.61)	(8.34, 8.61)	(8.17, 8.517)	(8.10, 8.34)	(8.21, 8.394)	
				XGB		89.10	89.42	87.15	90.84	89.37	615
					95% CI	(8.771, 9.140)	(8.752, 9.172)	(8.682, 8.925)	(8.940, 9.153)	(8.790, 9.041)	
5	Relief –F	Filter feature selection method	Histological type, tumor site, history of other cancers, age, vascular invasion, tumor size, type of treatment, tumor stage	SVM		83.82	82.16	81.92	84.61	82.93	1306
					95% CI	(8.241, 8.527)	(8.12, 8.417)	(8.034, 8.241)	(8.21, 8.516)	(8.124, 8.481)	
				HGB		82.47	83.61	82.56	81.62	82.31	1512
					95% CI	(8.170, 8.347)	(8.21, 8.492)	(8.17, 8.397)	(8.035, 8.306)	(8.094, 8.427)	
				XGB		83.75	84.30	82.07	83.92	81.01	1250
					95% CI	(8.201, 8.581)	(8.271, 8.609)	(8.092, 8.417)	(8.195, 8.463)	(8.037, 8.278)	

The full-features dataset and the features selected by the four feature selection algorithms were tested on three classifiers with the tenfold cross-validation method. In each fold, randomly 90% of the input vectors were chosen for training, and the remaining 10% were used for testing the models. To select the most important feature subset to predict the survival of patients with gastric cancer, the averages of five classifier performance metrics were calculated. Additionally, the full-features dataset was tested on the classifiers to compare the results with or without using feature selection methods.

Table 6 depicts the tenfold cross-validation results of four classifiers with the full-features dataset and eight variables selected by four feature selection techniques.

**Fig. 7:**
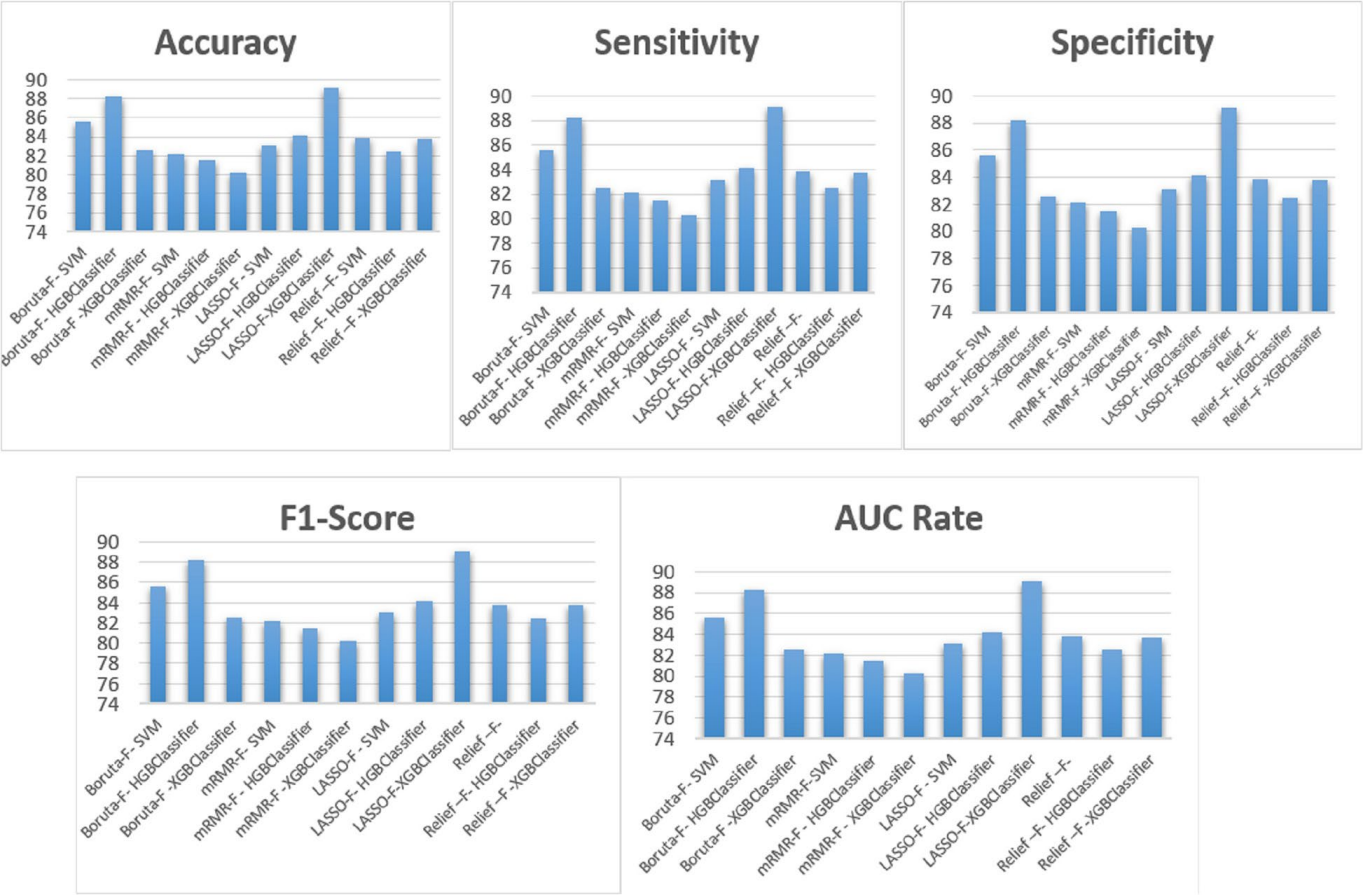
Average evaluation metrics of the classifiers using four different FS algorithms

**Fig. 8:**
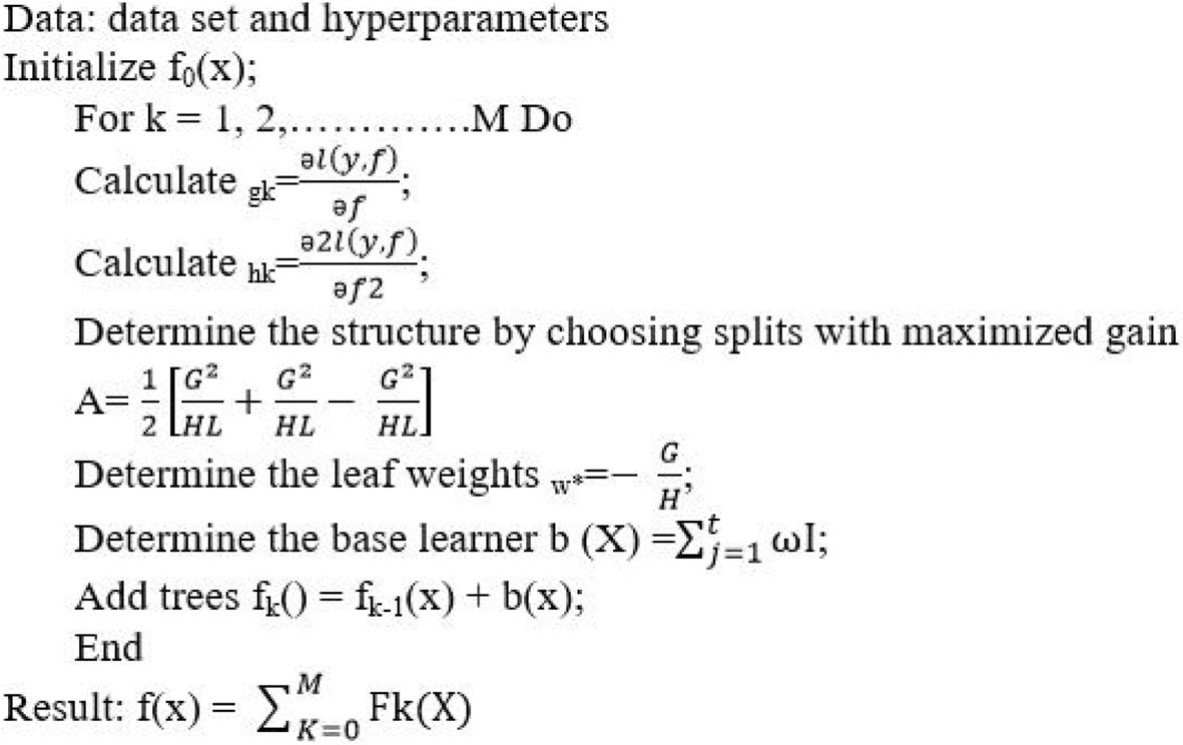
Pseudo-code of the XGBOOST algorithm to predict the five-year survival rate of gastric cancer patients

According to Table 6, when the full-features dataset was used for training the models, poor results were achieved. When the features selected by the FS algorithm were utilized, the LASSO feature selection algorithm combined with the XGB Classifier outperformed the other classification models. The LASSO algorithm combined with the XGB Classifier algorithms achieved 89.10% for average accuracy, 87.15% for average specificity, 89.42% for average sensitivity, 89.37% for AUC, and 90.8% for the f1-score value. As indicated in Table 6, the HGB classifier with the features selected by Boruta (a kind of wrapper-based technique) was the second-best model for gastric cancer survival prediction and scored 88.25% for average accuracy, 86.13% for average specificity, 89.71% for average sensitivity, 88.63% for AUC, and 89.31% for the f1-score. The best results for each evaluation metric are highlighted in Fig. 7. Considering the selected features by four FS algorithms, the most accurate prediction model was 89.07% obtained for the XGB Classifier when the LASSO algorithm was used. The pseudo-code of the XGB classifier is presented in Fig. 8. The highest rate for sensitivity, specificity, F1-score, and AUC metric was obtained for XGB Classifier and HGB Classifier, respectively.

The comparative accuracy of the classifiers before and after using feature selection techniques is given in Table 7.

**Table 7:** Accuracy of classifier before and after using feature selection techniques

Num	Classifiers	Best accuracy		Best AUC measurement	
		**Before features selection**	**After features selection**	**Before features selection**	**After features selection**
1	XGB	69.47	85.68 (by Boruta-F)	7.037	8.377(by Boruta-F)
2	HGB	62.58	88.25 (by Boruta-F)	6.206	8.863(by Boruta-F)
3	SVM	68.25	89.10 (by LASSO-F)	6.914	8.937 (by LASSO-F)

The time to build the model by the suggested approaches for optimizing prognostic variables of five-year survival in patients with gastric cancer is another crucial consideration. In the following, the effectiveness of each model is evaluated in terms of processing time, correctly categorized cases, incorrectly classified instances, and accuracy. The results are listed in Table 8.

**Table 8:** Time to build the model, correctly classified instances, incorrectly classified instances, and accuracy of the ML models

Feature selection algorithm used	NONE			Boruta-F			mRMR-F			LASSO-F			Relief –F		
Classifier	XGB	HGB	SVM	XGB	HGB	SVM	XGB	HGB	SVM	XGB	HGB	SVM	XGB	HGB	SVM
Time to build a model (s)	3081	3301	2849	2806	2930	2200	2624	2580	2147	2490	2028	1981	2924	1914	1280
Correctly classified instance	67	60	65	83	85	80	79	79	77	80	81	86	80	79	81
Incorrectly classified instance	30	37	32	14	12	17	18	18	20	17	16	11	17	18	16
Accuracy	69.47	62.58	68.25	85.68	88.25	82.54	82.12	81.46	80.24	83.07	84.12	89.10	83.82	82.47	83.75

Table 8 shows the results of classifiers in terms of model building time, correctly classified instances, and incorrectly classified instances. Table 8 demonstrates that the XGB Classifier, when the LASSO feature selection algorithm was employed, built its model in 1981s, making it the fastest; however, the HGB Classifier, when no feature selection approach was used, built its model in about 3301 s, making it the slowest.

## Discussion

This study compared various feature selection methods to determine the most effective predictor variables of five-year gastric cancer survival. Then, the selected variables were fed into three ML algorithms to develop predictive models. Finally, the models’ performance was compared to select the most optimal model for predicting five-year gastric cancer survival. This was done to minimize overfitting odds by not crowding the classifiers with too many input variables.

During the training process of ML techniques, it is necessary to use a huge amount of data samples to avoid overfitting [34]. However, it is not necessary to use a large number of data features to mitigate dimensionality [35, 36]. Furthermore, medical field data usually have interrelated and redundant features. Such features do not contribute any significant information to the prediction and also create noise in the description of the outcome variable, leading to prediction errors [37, 38]. Moreover, such features raise the intricacy of ML models and prolong their execution time. To deal with the problem of dimensionality, those features that affect the target variable should be identified as inputs to ML models [39]. The selection of effective features reduces the complexity of the models and, thus, increases their prediction accuracy [40].

Although the prevalence of gastric cancer has decreased, this disease is still the second cause of cancer deaths worldwide. Common classification systems such as the tumor, node, metastasis (TNM) staging system and traditional modeling based on statistical and mathematical methods are useful for classifying patients and modeling the risk factors influencing the onset and progression of the disease. Still, different and unbalanced variables with non-linear relationships affect gastric cancer, thereby complicating its prognosis and diagnosis. Therefore, the use of ML methods confers an added value.

This research attempted to use ML to predict the five-year survival of gastric patients and select the influencing features. The chief aim of this study was to observe the effect of feature selection methods on the performance of ML models. We experimented with four individual feature selection methods, covering all three of the basic categories, i.e., filter, wrapper, and embedded, and three ML algorithms.

In the feature selection phase, we observed that features such as tumor stage, tumor site, tumor size, and history of other cancers were ranked as the three top features by all the feature selection algorithms. The most significant variables for predicting survival among patients with gastric cancer were age, vascular invasion, type of treatment, weight loss, metastatic status, addiction, and lymphatic invasion, selected by one or more feature selection algorithms. Our findings are in agreement with those of some former studies, but there are still other clinical predictors which have been explored and selected by others. In the reviewed studies, after performing feature selection, many clinical predictors were determined as the important risk factors affecting gastric cancer survival. These variables were age [5, 41–43], sex [44–46], body mass index (BMI) [5, 45, 46], KPS [7, 41, 47], TNM stage [41, 43–46], tumor grade [6, 7, 41–43, 45, 46], tumor size [5, 6, 43, 45–47], tumor location [5, 6, 41–44], lymphovascular invasion [6, 7, 43, 45, 46], active and timely treatment [6, 7, 44], type of treatment [42, 43], disease stage and severity [5, 7, 41–44], and weight loss [43–45]. Thus, the results attained in the present study still need further investigation to select the most important and accurate predictors affecting gastric cancer. Future studies should analyze larger gastric cancer samples and include the features in our study.

The novelty of this study lies in developing models by comparing the performance of three ML algorithms for four feature selection methods to choose the best path for predicting five-year gastric cancer survival. To the best of our knowledge, this was the first study to compare the performance of several feature selection methods combining several ML algorithms on gastric cancer survival data Overall, our findings illustrate that ML methods can suggest more accurate alternates to classical statistical methods for survival prediction, particularly for dealing with high-dimensional datasets. The poor results obtained based on full-featured data might be due to overfitting problems.

So far, some efforts try to investigate the most influencing factors on the survival prognosis of patients with gastric cancer, using the extracted factors as the predictor inputs of ML models. Li et al. [48] retrospectively assessed the performance of SVM techniques to analyze the most prominent risk factors for gastric cancer survival prognosis. Ultimately, sex, carcinoembryonic antigen (CEA), lymph node metastasis, and protein expression were selected as important features. The SVM model achieved better performance with 85.17% accuracy and 0.93% AUC. Liu et al. [7] compared several ML techniques on the data of patients with gastric cancer to predict their survival. After evaluating different models, the gradient boosting algorithm had a higher performance with 84% accuracy. The variables such as the patient’s age, type of treatment, date of diagnosis, tumor characteristics, disease severity, disease metastasis, personal history, and history of timely and active chemotherapy were identified as the most important features affecting gastric cancer survival. Similarly, Akcay et al. [5] found that the patient’s age, cachexia, KPS score, treatment type (surgery or chemotherapy), tumor grade, location, and lymphatic invasion are the most significant factors to evaluate the overall survival (OS) by ML in gastric cancer cases. They found that XGBoost with an accuracy of 86% gained the best performance. Bang et al. [42] implemented different ML-based predictive models for gastric cancer; in their study, the XGBoost model was selected as the most efficient model with an accuracy of 93.4%. Nevertheless, the SVM classifier with an accuracy of 74.5% did not perform well in prediction. Diagnostic and clinical variables such as the date of diagnosis, the patient’s age at the time of diagnosis, disease severity, metastasis, histopathological type, history of gastric ulcer, the shape of the lesion, location of the lesion, and degree of gastric involvement were selected as important inputs to the ML models. On the other hand, the variables of sex, diet, weight, alcohol and drug consumption, and exercise were identified as less important risk factors. Fan et al. [44] found that the KPS, TNM stage, tumor grade, and metastasis status had a higher degree of importance as the inputs to ML models to predict lymphovascular invasion and survival of patients with gastric cancer in the early stages. In their study, after training different models, the Adaptive boosting (Adaboost) model achieved a higher performance with a 74.5% accuracy. Gao et al. [41] also reported that ML models, in particular SVM, would aid in active patient recurrence prognosis and survival prediction of gastric cancer cases (AUC range: 0.87–0.96). In their study, after performing feature selection, the variables of age, sex, BMI, KPS, TNM stage and cancer severity, tumor magnitude and position, metastatic, and treatment type were determined as the most important risk factors affecting gastric cancer survival. Chen et al. [43] also compared some data mining techniques for the survival prediction of patients with gastric cancer. The SVM algorithm with an accuracy of 78.9 achieved the best performance. Among 28 primary variables, invasion of the lymph nodes (lymphatic involvement), receiving active and effective surgical treatment, the stage and severity of the disease, and the amount of weight loss (cachexia) were selected as the most important variables.

The strength of this study lay in the possibility of comparing the feature selection methods along with ML classifiers in the survival analysis of gastric cancer data. Selecting the most influential factors in the survival of patients with gastric cancer was another advantage of the present research. However, there were some limitations that must be addressed. First, this study was conducted on a single-center dataset containing low-dimensionality data with only 17 features. In the future, more high-dimensionality feature datasets can be used to validate the proposed model. Moreover, multiple datasets of gastric cancer could be used and compared with the current results to improve the findings of this study. Second, four feature selection methods were employed to remove and rank the features. The proposed model can be modified by combining other feature selection techniques and/or using different ML methods to discard unrelated and redundant features. Finally, more classifier models beyond the applied ones can be combined to test, justify, and compare for better understanding.

## Conclusions

Biomedical data such as gastric cancer datasets are likely to have multifaceted, censored, varied, and mislaid values that challenge conventional statistical analysis. Therefore, computational techniques such as ML algorithms are required to overcome the challenges of analyzing these multidimensional data. The sample for gastric cancer prognosis data is very small; thus, feature selection methods are required to decrease the number of input features to circumvent the overfitting problem. Selecting the appropriately paired feature selection-classifier to predict the survival of gastric cancer patients can support the provision of personalized medicine, precise prediction, and selection of the proper treatment path.

## Data Availability

The datasets used and/or analyzed during the current study are available from the corresponding author on reasonable request.

## Abbreviations

ML: Machine learning
DT: Decision tree
SVM: Support vector machine
LASSO: Least absolute shrinkage and selection operator
mRMR: Minimum redundancy maximum relevance
XGB: XG Boost
HGB: Hist gradient boosting
Adaboost: Adaptive boosting
AUC: Receiver operating characteristic curve
HGB: Hist gradient boosting
CDSS: Clinical decision support system
WHO: World health organization
TNM: Tumor node metastasis
AI: Artificial intelligence
EMR: Electronic medical record
CRISP-DM: Cross-industry standard process for data mining
CI: Confidence intervals
ROC: Receiver operating characteristic
KPS: Karnofsky performance scale
